# Promises and trust in human–robot interaction

**DOI:** 10.1038/s41598-021-88622-9

**Published:** 2021-05-06

**Authors:** Lorenzo Cominelli, Francesco Feri, Roberto Garofalo, Caterina Giannetti, Miguel A. Meléndez-Jiménez, Alberto Greco, Mimma Nardelli, Enzo Pasquale Scilingo, Oliver Kirchkamp

**Affiliations:** 1 Department of Information Engineering and Center E. Piaggio, University of Pisa, Pisa, Italy; 2 Department of Economics, Royal Holloway University of London, London, UK; 3 Department of Economics and Management, University of Pisa, Pisa, Italy; 4 Department of Economic Theory and Economic History, University of Málaga, Malaga, Spain; 5 Chair of Behavioural and Experimental Economics, Friedrich-Schiller University Jena, Jena, Germany

**Keywords:** Biomedical engineering, Electrical and electronic engineering

## Abstract

Understanding human trust in machine partners has become imperative due to the widespread use of intelligent machines in a variety of applications and contexts. The aim of this paper is to investigate whether human-beings trust a social robot—i.e. a *human-like* robot that embodies emotional states, empathy, and non-verbal communication—differently than other types of agents. To do so, we adapt the well-known economic trust-game proposed by Charness and Dufwenberg (2006) to assess whether receiving a promise from a robot increases human-trust in it. We find that receiving a promise from the robot increases the trust of the human in it, but only for individuals who perceive the robot very similar to a human-being. Importantly, we observe a similar pattern in choices when we replace the humanoid counterpart with a real human but not when it is replaced by a computer-box. Additionally, we investigate participants’ psychophysiological reaction in terms of cardiovascular and electrodermal activity. Our results highlight an increased psychophysiological arousal when the game is played with the social robot compared to the computer-box. Taken all together, these results strongly support the development of technologies enhancing the humanity of robots.

## Introduction

Trust is considered as a social glue that connects people and promotes collective goals. It is normally defined as the “intention to accept vulnerability based on the positive expectations or beliefs regarding the intentions or behaviour of other people in general”^1^. As a consequence, behavioral science has always been interested in trust, and more particularly in its influence on decision making^2,3^. In parallel, trust is also relevant if we want to build social artificial agents that interact alongside people (e.g. robo-advisors, co-working robots, assistive robots, etc.) and take responsible roles in our society^4,5^. A lesson learned from previous research (e.g. economics, neuroeconomics, psychology) is that (general) trust is deeply rooted in social experiences, being more a matter of culture than genetics^1^, and highly affected by the emotional states of the individuals^6–8^. Indeed, emotions have been proven to play a fundamental role in the decision-making process in general^9^, as confirmed among other neuroscientists, by Damasio and colleagues in their studies^10–13^.

This stream of research thus suggests that trust and emotions are highly intertwined in the decision-making process in human–human interactions^14–17^, and may act as reasonable drivers in human–robot interactions as well^18^. It has been shown, for example, that not binding communications (i.e. cheap talk) is beneficial not only among humans but also to achieve higher cooperation when interacting with a machine (e.g^19^). In particular, a simple conversation with a robot changes individual attitude towards the artificial agent by making it appearing more like a social agent^4,20^. Very similar behavioural responses can be observed in children^4^. More in general, increasing the anthropomorphic features and the human social skills of a technology (e.g. by adding a name or a human voice to an autonomous vehicle) increases the individual willingness to accept and trust the technology itself (e.g.^13,21,22^).

Nonetheless, while the importance of emotions in driving the choice of a human to trust another human has been highly studied, less evidence is available when the decision to trust involves the interaction between artificial agents and humans (^7,21,23^). Moreover, we know that trust is highly culturally based, and that the appearance of the robot (especially its human-likeness, see^24^) affects the emotions perceived by its interlocutors. Therefore, studies on human–robot interactions and trust should always be repeated with different robot players having different aesthetics.

On that premise, the present study investigates how trust in a social robot is affected by its human likeness (both in terms of aesthetics and speech content), while taking into account the psychophysiological states of the players during the interaction through physiological signal processing. The objectives are twofold. On the one side, we can gain insights into how human-likeness interacts with emotions to instill people’s trust in artificial agents, comparing it with that in human partners so as to assess the differences (if any). On the other side, we can gain a better understanding on how to design machines—both in terms of appearance and (e.g. communication) skills—in a way that helps facilitate a fruitful interaction with humans. To this end, we present a series of experimental conditions based on a modified version of a well-known game used in behavioral economics to study trust among humans: the trust game as proposed by Berg and colleagues and adapted by Charness and Dufwenberg^25,26^. In this game, the outcome of the interaction depends on whether the first mover (the trustor) decides or not to trust the second mover (the trustee). If the first mover decides to trust the counterpart by remaining in the game, the second mover has to decide between a choice that does not benefit the trustor but it is more beneficial for himself (i.e. provides him with the highest payoff) and a choice that benefits the trustor but provides him with a lower payoff. If the first mover decides not trust, both players get a lower outside payoff. In other words, there is a conflict of interest between the two players when remaining in the game, but both would be better off if a mutual relationship is established (i.e. the first player remains in the game). A peculiar characteristic of this game is that prior to the trustor’s choice of remaining in the game, the trustee is given the opportunity to send him a non-binding (i.e. cheap-talk) message. We rely on this game as it has been specifically conceived to assess whether receiving a message containing a promise from the opponent increases individual trust in him (her).

**Figure 1:**
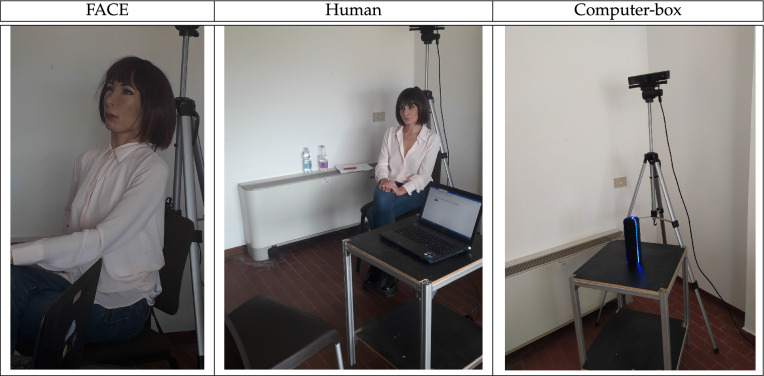
Three types of player-B.

In our experiment the role of the trustor is always played by a human participant while the role of the trustee is played by three different types of players: a humanoid robot with high human-likeness (*F*ACE, Fig. 1), a human counter-part (*Human*, Fig. 1), and a computer-box machine (*Computer-Box*, Fig. 1). In all cases, we compare the trustors’ choices when the trustee sends a generic message—not including any type of promise (i.e. an ‘*empty*’ message)—with the trustors’ choices when the trustee sends instead a message containing a promise. Specifically, to generate the messages from the robot, we rely on real sentences that occurred between human participants in the experiment of Charness and Dufwenberg^25^, and were therein classified either as empty or promising. In addition, to monitor the psychophysiological states of our participants, throughout all the experimental conditions we collect data on the most widely used autonomic nervous system correlates (ANS), such as pulse rate variability (PRV) and electrodermal activity (EDA), which are well known to contain information about the affective state of a subject^27^. PRV represents the variation in the time interval between two heartbeats, whereas EDA measures changes in skin conductance due to psychologically-induced sweat gland activity. They were measured on the wrist surface through a sensorized bracelet (i.e., Empatica’s E4 wrist band).

## Experimental design

**Figure 2:**
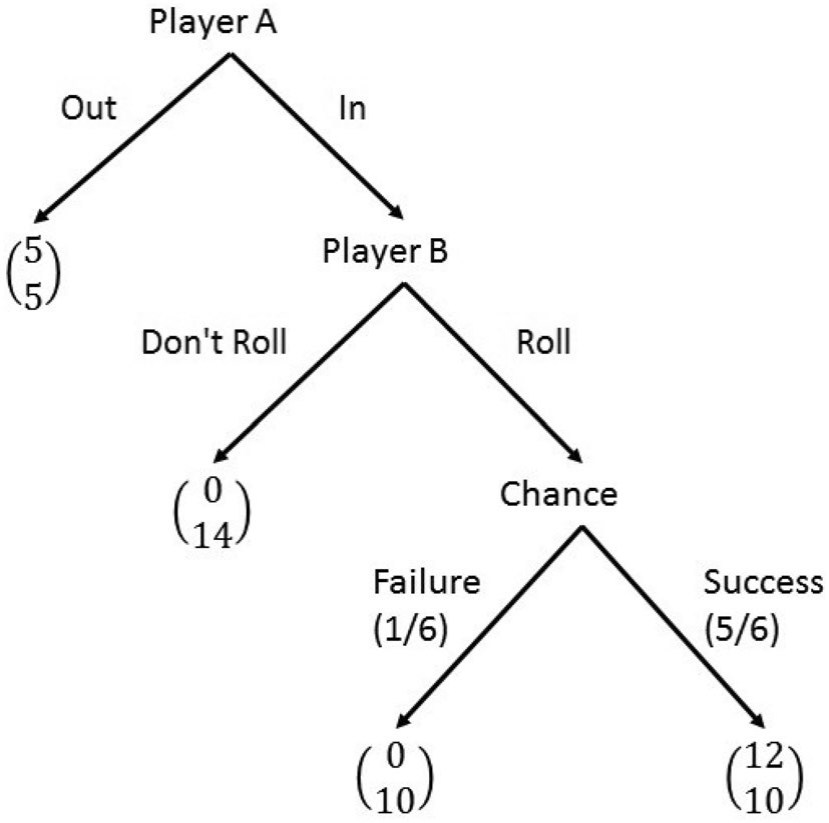
The game.

In the experiment we replicate the trust game proposed by Charness and Dufwenberg^25^ as depicted in Fig. 2. There are two players: A (the trustor) and B (the trustee). Player-A chooses between two options,* In* and *Out*. If Player-A chooses *Out*, the game ends and each player wins 5 Euro. If Player-A chooses* In,* then Player-B has to choose between two options, *Roll* or *Don’t Roll*. If he chooses *Don’t Roll*, then he wins 14 Euro while Player-A earns 0 Euro. If he chooses *Roll*, Player-A wins 0 Euro with probability 1/6 and 12 Euro with probability 5/6, while Player-B wins 10 Euro in any case. From an economic point of view, for Player-B it is better if Player-A chooses *In*, while for Player-A choosing *In* is convenient only if B chooses *Roll*. A characteristic of this game is that when Player-A wins 0, it is not possible for Player-A to infer with certainty whether Player-B has chosen either *Roll* or *Don’t Roll*. This game thus reflects (as many other experiments in economics) real-world situations where it is not possible to perfectly observe the behaviour of a partner that can be delegated to make relevant payoff decisions. In this experiment, the type of Player-B (i.e., the trustee) changes across experimental conditions, while Player-A is always a human participant. In particular, the role of Player-B is played by either a humanoid (FACE), a computer-box, or a human. Regarding the message Player-B sends to Player-A, it can be of two kinds: a message containing a promise to roll the dice *(promising)*, and a generic message *(empty)*. In particular, we select messages from the original study of Charness and Dufwenberg^25^ (as available on the related Supplementary material in the online appendix). To further check whether the length of messages affects individual choices, for each type of message (i.e. promising and empty), we specifically select two short (less than 10 s) and two long (more than 10 s) messages. Thus, we have a 3x2x2 design. Experimental conditions are illustrated in Table 1 and 2, and an English translation of the instructions is available in the last section at the end of the paper.

In the FACE condition, the role of Player-B is played by FACE, i.e. a hyper-realistic humanoid robot with the aesthetics of a woman (see Fig. 1) that due to its perceptive, reasoning, and expressive capabilities, constitutes a sophisticated observation platform to study what happens when human and machine establish empathic links^28,29^. However, although it has been shown that humanoid robots can use the expression of emotion to influence human perceptions of trustworthiness, we do not rely on FACE’s ability to show emotional information through facial expressions in order to isolate only the effect of human-likeness and promise in influencing the emotional state of our participants, as well as their choices.

**Table 1:** Experimental conditions.

	Empty			Promising			Grand total
	Short	Long	Total	Short	Long	Total	
Computer-box	12	19	31	20	13	33	64
Human	16	10	26	14	8	22	48
Humanoid (FACE)	15	10	25	16	9	25	50
Total	43	39	82	50	30	80	162

This table classifies the number of observations collected in our study according to the type of counterpart the human participants confront with (i.e. computer-box, human, and humanoid) and the type of sentence they have to listen to (i.e. cointaining a promise or not, either a short or long sentence).

In the *Computer-Box* condition, the role of Player-B is played by a light-emitting audio-box reproducing the same audio-sentences and taking decisions in the same way as in FACE. Importantly, both in *FACE* and *Computer-Box* conditions, the artificial agent has its own cognitive system with its perception analysis and architecture, i.e. the so-called Social Emotional Artificial Intelligence (SEAI). This framework allows the social scenario to be acquired and to influence the parameters which correspond to the ‘mood’ of the artificial agent (see and ^30^). Specifically, in this experiment, due to SEAI, the artificial agent benefits from its own artificial emotions for choosing whether to *Roll* or *Don’t Roll* (see the section How the robot takes a decision, the rules engine in Methods for more information about how the robot takes a decision). More importantly, the participants in this experiment are aware that the artificial agent (like the human counterpart) is able to take its decision autonomously, i.e. not randomly but following its own behavioural rules, and therefore the results of game interaction are not determined by chance only.

In the *Human* condition, the role of Player-B is played by the same professional actress who gave her voice for recording FACE/Computer-Box’ audios. The actress is free to autonomously decide her choices in the game, i.e. *Roll* or *Don’t Roll*, being paid accordingly, but she has no room to decide which sentences to state that have to be exactly the same ones, and in the same identical order, as the ones pronounced in *FACE* and *Computer-Box*. Moreover, the actress is instructed to avoid any facial expressions during the interaction with a participant, and has to wear FACE’s hair and dresses. Similarly, she has to follow the same experimental procedure as in the Computer-Box and FACE conditions (see the section Experimental procedure in Methods for details on the experimental procedure).

To investigate the psychophysiological state of Player-A while taking the decision, in all experimental conditions the participants wear a wearable device on their left wrist (a sensorized bracelet, the Empatica’s E4 wristband) for the real-time collection of physiological data, such as PRV and EDA. The processing of these signals allows us to characterize the ANS activity of Player-A and infer about his (her) psychophysiological states. In particular, to quantify the autonomic nervous system activity we extracted three indexes to quantify both the sympathetic branch (i.e. the EDAsymp index^31^), the parasympathetic branch (i.e., the HFnu index^32^), and the sympthovagal balance (i.e. EDAHFnu index^33,34^). In sections Description and analysis of physio data and New index from the sympathovagal assessment in Methods we describe in details how we computed these indexes.

At the end of the experiment, participants have to fill in a questionnaire asking information about how they perceive Player-B, as well as information about their individual characteristics, such as age, gender, and field of studies. In particular, as Nitsch and Glassen^20^, participants has to rate on 7-likert scale how much they perceive Player-B as a human (i.e. the human-likeness, where 1 means non-human at all and 7 means totally human) and how much they perceive Player-B as a machine (i.e. the machine-likeness). We also ask participants to rate how much they believe their behaviour has affected Player-B’s choice and to make a guess about Player-B’s choice (Roll/Don’t roll). Finally, we elicit their technological affinity by the Affinity for Technological Interaction (ATI) scale as in Franke and coauthors^35^ and measure their individual risk preferences with the International Test on Risk Attitudes (INTRA tests^36^).

The experiment was conducted from the end of July till October 2019, and 162 randomly invited participants out of a pool of more than 1500 students coming from all departments of the University of Pisa took part in the study (90 students were female and 72 male with no substantial difference across experimental conditions). For more information on the protocol see the section Participants in Methods at the end of the paper.

**Table 2:** Type of messages.

Types	# phrases	# seconds	phrases
Empty	2	< 10	*’Good luck!’*
			*‘Please choose IN, so we both earn more money.’*
	2	> 10	*‘If you stay IN, the chances of the die coming up other than 1 are 5 in 6 – pretty good. Otherwise, should you choose OUT we’d both be stuck at 5 Euro.’*
			*‘Good luck on your decision. Choose whatever. If you choose “out”, you get only 5 Euro more. If you choose “In” you can get 12 Euro instead of only 5 Euro. 7 Euro more is a lot of money!’*
Promising	2	< 10	*‘I will roll the dice’*
			*‘Choose In and I will Roll. You have my word.’*
	2	> 10	*‘Choose in, I will roll dice, you are 5/6 likely to get 2,3,4,5, or 6 and win 12 Euro. This way both of us will win something.’*
			*‘Choose in and I will roll. That way, we’ll both get extra money.’*

This table reports 8 sentences that occured between human participants in the study of Charness and Dufwenberg (2006) and were selected in our study. 4 out of 8 sentences were classified as short (i.e. they last less than 10 s) and empty (i.e. they did not contain any type of promise to roll the dice).

## Results

We start analyzing how participants rated the different types of player-B as a human and a machine, as well as their technological affinity. In Table 3 we report the average of these variables by type of Player-B. Note that in the following, we denote with *p*_*p*_ the one-sided *p* value for a test for proportions, with *p*_*t*_ the one-sided *p* value for a t Student test, and with *p*_*perm*_ the one-sided *p* value for a test with 500 data permutations (see more information in the section Mean comparisons across groups in Methods). If we compare how much individuals rated Player-B as a human, we observe that *Human* is ranked higher than *Face* (mean diff = 1.49, *p*_*t*_ = 0.000), and *Face* is ranked higher than *Computer-box *(mean diff = 0.87, *p*_*t*_ = 0.007). Moreover, if we look at how participants assessed Player-B as a machine, we consistently find that *Face* ranked higher than* Human* (mean diff = 2.03, *p*_*t*_ = 0.000). It is important to remark that we ask our participants to give the same rating also to the human (actress) counterpart as her behaviour is not entirely natural, as she has to avoid any additional interactions as well as any facial expression during the game. We do not find any significant difference in technological affinity between participants in the different experimental conditions.

**Table 3:** Participants’ perception and technological affinity.

	Human-likeness	Machine-likeness	ATI
*Human*	4.96	3.60	4.84
*FACE*	3.46	5.64	5.08
*Computer-Box*	2.59	5.93	4.98
Total	3.56	5.15	4.97

For each type of player-B, this table reports the average values of variables measuring on a scale from 0 to 7 human-likeness, machine-likeness and technological affinity (ATI scale as in^35^).

The main results are summarized in Table 4, which reports the relative frequencies of choice ‘In’ made by participants (acting as Player-A) by experimental conditions and human-likeness. Specifically, for each type of Player-B, we categorize the level of human-likeness as *Low* when the participant rating is below the median choice (on the distribution on the 7-likert scale), and *High* otherwise. Note that we pool the data regardless the length of the message, since it does not significantly affect the decisions to play ‘In’ in any scenario.

We first compare the results according to the type of Player-B. We note that the frequency of choice ‘In’ is significantly lower when player-B is a Human than when player B is either FACE (0.60 vs. 0.80, mean diff = − 0.20, *p*_*p*_ = 0.030, *p*_*perm*_ = 0.016) or a Computer-box (0.77, mean diff = − 0.17, *p*_*p*_ = 0.066, *p*_*perm*_ = 0.016). There is no significant difference between FACE and Computer-box.

Regarding the effect of receiving a promise (vs. receiving an empty message), we do not find any significant effect on the frequency of choice ‘In’ looking at each type of player-B separately. However if we distinguish by human-likeness, we find significant effects of receiving a promise. Specifically, when Player-B is Human and human-likeness is high, the frequency of choice ’In’ is significantly higher when a promise is received (0.86 vs. 0.53, mean diff = 0.33, *p*_*p*_ = 0.030, *p*_*perm*_ = 0.018). A similar suggestive evidence, is found when Player-B is FACE and human-likeness is high (1 vs. 0.85, mean diff = 0.15, *p*_*p*_ = 0.097, *p*_*perm*_ = 0.000).

**Table 4:** Relative frequencies of ‘choice in’ by experimental condition and human-likeness.

	Human-likeness		Total
	Low	High	
**FACE**			
Empty	0.67	0.85	0.76
	[12]	[13]	[25]
Promising	0.73	1	0.84
	[15]	[10]	[25]
Total	0.70	0.91	0.80
	[27]	[23]	[50]
**Human**			
Empty	0.55	0.53	0.54
	[11]	[15]	[26]
Promising	0.37	0.86	0.68
	[8]	[14]	[22]
Total	0.47	0.69	0.60
	[19]	[29]	[48]
**Computer-box**			
Empty	0.71	0.80	0.74
	[21]	[10]	[31]
Promising	0.79	0.79	0.79
	[19]	[14]	[33]
Total	0.75	0.79	0.77
	[40]	[24]	[64]

This table reports the relative frequencies of (i.e. the share of participants) choosing ‘IN’ for each experimental condition by human-likeness. Human-likeness is Low when the participant rating is in the lower side of the distribution on the 7-likert scale, and High otherwise. The number of observations are in squared brackets.

We now delve into the effects of human-likeness for each type of Player-B. To begin with, we observe that if participants assigned a high human-likeness to Player-B, the probability of choosing ‘In’ is significantly higher than those who assigned it a low human-likeness when Player-B is either FACE (0.91 vs. 0.70, mean diff = 0.21, *p*_*p*_ = 0.033, *p*_*perm*_ = 0.010) or Human (0.69 vs. 0.47, mean diff = 0.22, *p*_*p*_ = 0.067, *p*_*perm*_ = 0.032). There is no significant difference when Player-B is a Computer-box. Furthermore, if we distinguish between the group of participants who received a promise from those who received an empty message, we observe that, when Player-B is FACE, the effect of higher human-likeness is significant only among those who received a promise (1 vs. 0.73, mean diff = 0.27, *p*_*p*_ = 0.037, *p*_*perm*_ = 0.000). A similar result is observed when Player-B is Human (0.86 vs. 0.37, mean diff= 0.49, *p*_*p*_ = 0.010, *p*_*perm*_ = 0.002). Overall, we can conclude that the choice to trust FACE is significantly related to the way a participant perceived it as a human. If a participant recognises FACE very similar to a human being, the probability that he will choose ‘In’ increases. We find that this effect is mainly driven by those participants who received a promise. This result may be rationalized in terms of a simple behavioral model that takes into account the possibility of aversion to lying, a feature that is commonly used to explain behavior in the literature of behavioral/experimental economics (^37,38^). See section A stylized behavioral model in the Methods for details.

**Figure 3:**
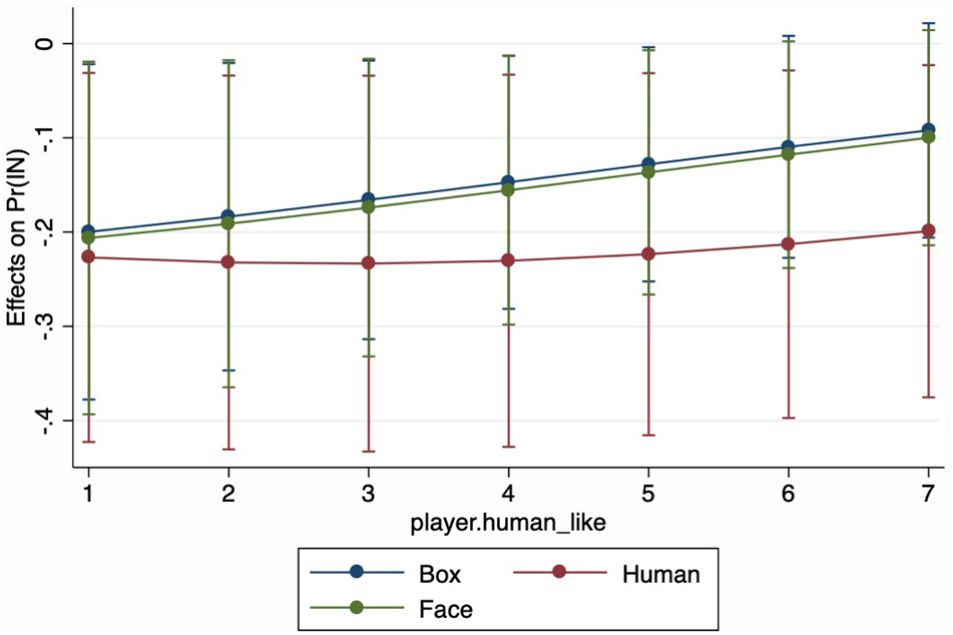
Marginal effect of Sympamp High on the probability of playing ’In’.

If we attend to the emotional reaction of the participants, we need to caution about possible over-segmentation of our data in the analyses that follow. It is important to acknowledge that we lose some observations for what concerns the measurements of the psychophysiological parameters for Computer-box (20 obs) and Human (25 obs) due to noise in the data, resulting in a smaller sample size. However, in most comparisons that we run, the number of observations in each cell is always above 8/10. In any case, as before, we additionally use statistical tests that do not rely on any specific type of distributions (and thus suffer less of a smaller sample size). Furthermore, we verify the robustness of the results by changing the time-window of our analysis. In all cases results appear robust. On top of that, in the final analysis depicted in Fig. 3, we rely on the continuous variable for human-likeness, without segmenting our dataset.

**Table 5:** Physiological data: EDAsymp and EDAhf_nu.

Index	Human-likeness	*Box*	*Human*	*FACE*
EDASymp	LOW	− 0.144	− 0.288	− 0.129
		[28]	[9]	[26]
	HIGH	− 0.327	− 0.128	1.731
		[16]	[16]	[22]
	Total	− 0.211	− 0.186	0.724
EDAHFnu	LOW	− 0.175	− 2.173	0.275
		[28]	[9]	[26]
	HIGH	0.012	0.055	5.865
		[16]	[16]	[22]
	Total	− 0.107	− 0.747	2.837

The EDAsymp index quantifies the activity of the sympathetic nervous system, while the EDAHFnu index quantifies the sympthovagal balance. A full description is available in the sections Description and analysis of physio data and New index from the sympathovagal assessment in Methods. Human-likeness is Low when the participant rating is in the lower side of the distribution on the 7-likert scale, and High otherwise. The number of observations are in squared brackets.

Having said that, in the following, we concentrate on two out of the three indexes computed using the physiological data recorded during the experiment, namely EDAsymp and EDAHFnu (see Table 5), as the third index HFnu provides only suggestive—although consistent—evidence. Specifically, we find a significantly higher autonomic nervous system (ANS) activation when Player-B is FACE that when Player-B is either Computer-box (0.724 vs. − 0.211, $\hbox{mean diff}_{EDAsymp}=0.935$, *p*_*t*_ = 0.016, *p*_*perm*_ = 0.008; 2.837 vs. − 0.107, $\hbox{mean diff}_{EDAHFnu}=2.944$, *p*_*t*_ = 0.053, *p*_*perm*_ = 0.050) or Human (0.724 vs. − 0.186, $\hbox{mean diff}_{EDAsymp}=0.909$, *p*_*t*_ = 0.056, *p*_*perm*_ = 0.074; 2.837 vs. − 0.747, $\hbox{mean diff}_{EDAHFnu}=3.584$, *p*_*t*_ = 0.063, *p*_*perm*_ = 0.068). Furthermore, when Player-B is FACE, we find that subjects who rated Player-B high in human-likeness are more likely to experience a stronger emotional reaction than participants who rated it low (1.731 vs. − 0.129, $\hbox{mean diff}_{EDAsymp}=-1.859$, *p*_*t*_ = 0.017, *p*_*perm*_ = 0.000; 5.865 vs. 0.275 $_{EDAHFnu}=-5.590$, *p*_*t*_ = 0.009, *p*_*perm*_ = 0.000). We do not find a similar effect when Player-B is Human or Computer-box. Finally, we note that the psychophysiological reaction of subjects rating FACE high in human-likeness is significantly higher than that experienced by subjects interacting either with Computer-box or Human, regardless of the rating of human-likeness.

Regarding the relationship between the psychophysiological reaction of participants and their choices, we do not find any significant correlation using the two indexes EDAsymp and EDAHFnu. However, if we split our participants into two groups according to whether they express a stronger (or weaker) psychophysiological reaction than the median level of the distribution of EDAsymp (see Table 6), we can observe that those who experienced a stronger reaction are also less likely to choose ’In’ in both Computer (0.636 vs. 0.909, mean diff = 0.273, and *p*_*p*_ = 0.015) and Human (0.462 vs. 0.750, diff = 0.288, and *p*_*p*_ = 0.070.

**Figure 4:**
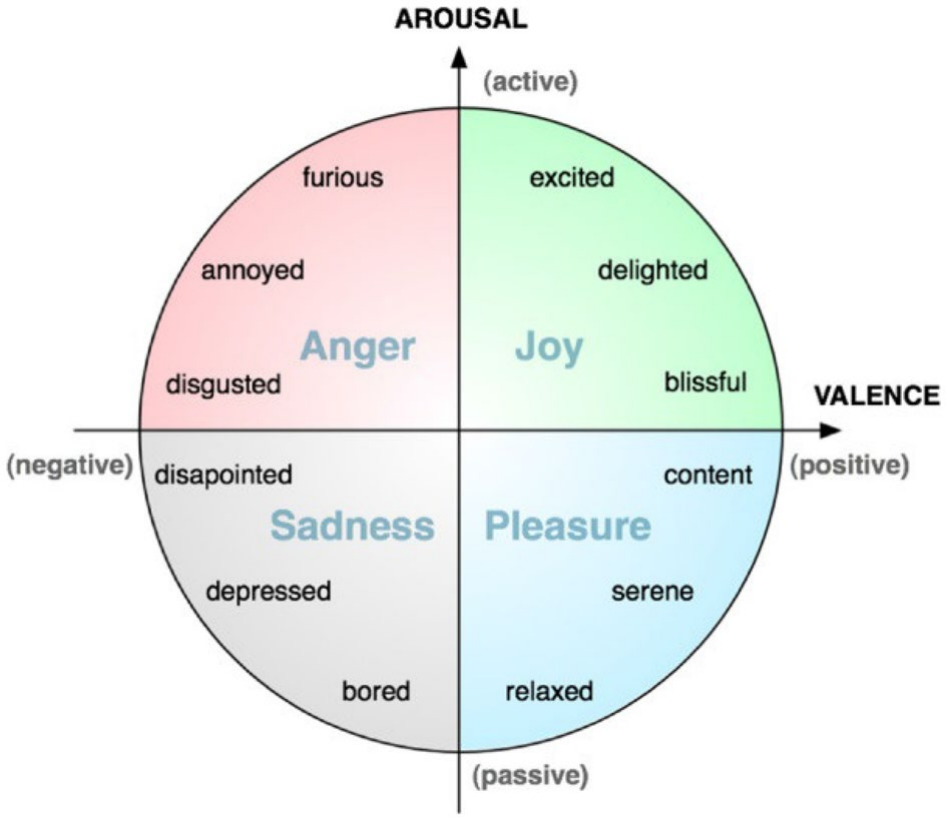
Emotional state of the robot.

Finally to study the interaction between human-likeness and psychophysiological reaction of our participants we conduct a probit analysis for the probability of playing ‘In’ using as a set of regressors player human-likeness and EDAsymp dummy, along with a dummy for each experimental conditions. Results are reported in Fig. 4. This figure highlights that increasing the psychophysiological reaction from low to high reduces the probability of playing ’In’. However, increasing the level of human-likeness counterbalances this negative effect, especially in FACE and in Computer-box.

**Table 6:** Relative frequencies of ‘choice in’by physiological state and human-likeness.

Human-likeness	EDASymp		Total
	High	Low	
**FACE**			
High	0.916	0.900	0.909
	[12]	[10]	[22]
Low	0.667	0.714	0.692
	[12]	[14]	[26]
Total	0.792	0.792	0.792
	[24]	[24]	[48]
**Computer-box**			
High	0.667	0.857	0.750
	[7]	[9]	[16]
Low	0.616	0.933	0.786
	[15]	[13]	[28]
Total	0.636	0.909	0.770
	[22]	[22]	[44]
**Human**			
High	0.500	0.875	0.686
	[8]	[8]	[16]
Low	0.400	0.500	0.444
	[5]	[4]	[9]
Total	0.462	0.750	0.600
	[13]	[12]	[25]

Each cell represents the frequencies of choice ‘In’ within each category. An individual is classified in EDAsymp High whenever is above the median level of the EDAsymp distribution, and EDAsymp Low otherwise. Human-likeness is Low when the participant rating is in the lower side of the distribution on the 7-likert scale, and High otherwise. The number of observations are in squared brackets.

## Discussion and conclusion

In our experiment participants were confronted with a counterpart which differed in the degree of human-likeness: a light-emitting computer-box, a female humanoid and a female human (which resembled the humanoid). The participants needed to decide—after listening to a message from the counterpart, containing in half of the cases a promise—whether to trust or not their opponent in the game. We find evidence that a human receiving a promise from a humanoid has more trust in it only when he (or she) perceived the artificial agent very similar to a human-being. Indeed, if we replace the social robot with a human we find a similar pattern. However, replacing it by the computer-box the effect of receiving a promise disappears. We also find that participants experienced a stronger psychophysiological reaction when confronted with a humanoid, especially if it appeared to them very close to human. Moreover, we observe that those participants expressing stronger psychophysiological reaction were less likely to trust the counterpart (i.e. chose more often the safer option) when this is either a computer-box or a human.

Taken all together, these results suggest that human-likeness and (integral) emotions play both an important role in the decision to trust the counterpart, possibly in interaction with each other. However, some remarks follow in order. While in this experiment we can fully control the degree of human-likeness by varying it across experimental conditions, we have less control over the type of emotions experienced by our subjects. Although physiological measures such electrodermal activity (EDA) have been widely used over the last decades for representing emotional arousal, and most scholars accept a physiological component in the definition of emotions, it is not possible to directly match the physiological state of a participant with a direct type of emotion (e.g. fear or anxiety). In addition, the literature on emotion arousal highlights that there might be individuals exhibiting different physiological responses to the same emotional state^39^. Therefore, our results can only suggest a greater or a weaker ‘emotional arousal’ without giving any insights into the type of emotions proved by our participants.

Nevertheless, the vast psychological literature on emotions and decision-making offers us an interesting framework to interpret our results. In particular, recent evidence from laboratory experiments is mostly consistent with the Appraisal-Tendency Framework according to which emotions change individuals’ appraisal of a situation, thereby affecting individual choices^9,40^. Importantly, in that framing, emotions of the same valence (such as fear and anger) can exert opposing influences on choices. Thus, what matters is whether an emotion (either positive or negative) by leading to a more cautious appraisal of the situation reduces the feeling of control, e.g. thereby reducing the willingness to take risks. Therefore, even if we are not able to disentangle among different types of emotions, we can reasonably assert that in our framework, whenever the experience of a stronger emotional arousal lead a participant to a more cautious appraisal of the counterpart, we observe a more careful assessment of the situation and a lower willingness to take risk and trust the counterpart. This interpretation of our results is also consistent with previous research showing that participants with ventromedial prefrontal cortex (a key area of the brain for integrating emotion and cognition) repeatedly select a riskier financial option over a safer one, even to the point of bankruptcy, despite their understanding of the suboptimality of their choices. In particular, their physiological measure of skin response suggests that they did not experience the emotional signals (i.e. the somatic markers) that lead normal decision makers to fear high risks^9^. This result is also in line with the recent work of Schniter and co-authors^7^, who similarly find that the emotional reactions of playing a trust-game against a human are substantially different from those arising with playing against a computer robot.

However, we must also notice that our results in terms of trust choices hold within a very specific setting, in which the human counterpart has been a bit ‘dehumanized’. It would be interesting to study whether relying on a large variety of human opponents, we still observe that a greater level of human-likeness is associated with higher trust. Indeed, studies of dehumanization show that there is a series of characteristics (e.g. socialibility or warmth) that are perceived as critical for the perception of others as human^41^, and it might well be the case that only a specific subset could be relevant for trust choices.

In addition, it would be extremely interesting to see whether trust translates into subsequent rounds, therefore extending the results from our simple one-shot game to repeated interactions and over a longer time-horizon. In that sense, it would be worthwhile to study the differences (if any) between being betrayed by a human rather than by a humanoid or a computer.To sum-up, we believe that our results strongly support the efforts in developing technologies enhancing the humanity of social robots, both in terms of human appearance and communication behaviour. Indeed, if from one-side it is not possible to control for human emotions, in line with recent studies^21,22^, our results suggest that increasing the human-likeness of an artificial agent increases sensibly the likelihood that a human counterpart will trust it as well as the associated emotional response.

To conclude, we see several directions for future interdisciplinary research. The first one is to explore different types of human–robot interactions, for example, prisoner dilemma games, coordination games or repeated interactions (e.g. by replicating the analysis of Crandall and co-authors with a social robot^19^). The second direction of research is on the side of the social robot. To keep the design as clear as possible, we did not rely on the humanoid’s ability to show facial expression. It would be very interesting to introduce within this setting the possibility of the robot to adapt its facial expression, as well as the mode of communication, to the perceived emotions of the human counterpart. In this way, we could actually test whether a greater ability to express humanness still lead to higher trust.

## Methods

### Participants

The experimental protocol was approved with unanimity by the Bioethical Committee of the University of Pisa (Review No. 21/2019), and all experimental conditions were conducted in accordance with relevant regulations and guidelines. Informed consent was obtained from all participants in the experiments, including from the actress so as to publish online her picture reported in Fig. 1.

Participants were invited through ORSEE system of the University of Pisa, which allow to randomly invite participants and to keep track of their participation in experiments over time^42^. In total 164 participants signed-up and showed up in the laboratory in the day they were invited. Two subjects were removed from the pool because they did not follow the experimental procedure correctly. The final sample was therefore of 162 (90 students were female and 72 male, with a mean age of about 26 years old).

The total number of participants to recruit was determined based upon the study of Charness and Dufwenberg 2006, as well as taking into accountour technical constraints (i.e. the impossibility to run the humanoid for a long period of time in a day). More specifically, in Charness and Dufwenberg (2006) there were 42 pairs in the experimental condition in which participants could receive a message from the opponent B, with a share of 0.74 of player-B actually choosing ’In’. We knew that given the proportion of 0.74 in the Human condition, the smallest difference that could be detected with this sample size and a power of 0.80 was about 0.20. Therefore, we aimed to have a final sample of about 50 participants (i.e. having 50 pairs for each experimental conditions), thereby inviting 55 participants for each experimental condition (to account for having some participants not showing up). In the Computer-box we decided to invite more than 55 participants as several participants did not no-show up in the previous days, ending up with a slightly higher number of subjects for this experimental condition compared to the other two. Of note, our research has been conducted following an exploratory approach lacking in the literature strong and reliable evidence on which to ground our hypotheses. Consequently, the paper has not been preregistered.

### Experimental procedure

Each participant arrives in the laboratory and enters a room in which (s)he is invited to read and sign the consent to participate in the study. The participant then sits in front of a computer screen where (s)he can read autonomously the experiment instructions and fill in some preliminary questions, such as own attitudes towards the technology. At this stage, the participant has to wear the bracelet ’Empatica’ on the left wrist, (as this phase will then be used as ‘the rest’ phase for measuring psychophysiological parameters (see also below section 4.7). Once the participant has completed this part, the participant is lead by the experimenter to another room where player-B (i.e. either the human, the humanoid or the computer-box) is located: just before entering this new room a marker is recorded on the bracelet to begin the second phase of measurement of psychophysiological parameters. The participant sits on a chair, always located at the same distance from player-B, and when ready to start the experiment has to raise the right hand. At this point, player-B welcomes the participant with a standard sentence (‘Nice to meet you! Let’s start’) to then state one random sentence out of 8 (according to the experimental condition, see again Table 2 in the paper). Player-B then tells the participant a standard final sentence, inviting the participant to enter his(her) choice in the computer in front of him(her). Importantly, player-B can never observe the choice the participant has made. To conclude the experiment, the participant has to return to the initial room, to complete an exit questionnaire about the interaction with player-B, and receive the final payment.

### The FACE robot and the SEAI cognitive system

The FACE robot (Facial Automaton for Conveying Emotions) is a humanoid with hyper-realistic adult female aesthetics, specifically designed for social robotics^43^. It is composed with a passive body on the top of which a Hanson Robotics’ head has been mounted. The head is designed to host 32 servomotors that guide the neck of the robot, its eyes, mouth, and facial expression. The face of the ginoid is made of Frubber (https://patents.google.com/patent/US7113848?oq=frubber) a registered material with skin-like mechanical and aesthetical features. This hardware is controlled by SEAI (Social Emotional Artificial Intelligence), a distributed control architecture made of perception, cognitive and actuation systems, that endow the robot with expressive and communicative capabilities^30^, including also the possibility to emulate verbal communication following prerecorded audio files. The audio files used for the experiment have been recorded using the voice of a professional actress, the same who interpreted the role of Player-B in the interactions with the real person; the sentences were the Italian translation of the sentences between the Charness trust game players. SEAI is a bio-inspired architecture based on neuroscientific theories of mind. In particular, it has been inspired by the findings of Antonio Damasio and it is consistent with the computational formalization made by^44^. In its development, the influence of emotions in the decision-making process has been of primary importance. The perception part of the system is the Scene Analyzer, an audiovisual perception system conceived to analyze a social environment using the robot sensors and to extract meaningful social cues from these available data. Features that can be extracted from a human interlocutor are, e.g., the three dimensional position of 25 joint coordinates, their speaking probability, meaningful postures and gestures, estimated facial expressions, age and gender^45^. This Social Perception System has already been successfully integrated with the acquisition of physiological parameters (i.e., electrodermal activity, respiration rate and heart rate variability) in past experiments^46^. All the environmental information anlayzed by the perception system of the robot is then processed by its cognitive system, i.e., the I-CLIPS Brain^47^, a rule-based expert system written in CLIPS language^48^. The knowledge base of the expert system is written by means of IF-THIS-THEN-THAT rules, where each rule contains a set of actions that will be executed if several conditions about the upcoming factual information are satisfied. Thanks to these rules it is possible to design the behavior of the humanoid. For example, a particular expression gathered in its interlocutor can lead to the trigger of a sentence or a facial expression performed by the robot, but also to the modification of the robot’s internal values. In fact, SEAI includes emotional internal values (i.e., valence and arousal), which combination describes an emotional state, here defined as *mood* (see Fig. 4). This method of representing emotion is based on the well-known Russell’s Circumplex Model of Affect^49^. In the case of the robot, mood is not necessarily externalised by perceivable movements, rather it is implied in biasing the chaining of the rules, and so, the decision tree of the robot. Emotion biasing decision in this cognitive system has been previously tested^50^. The instructions coming from the cognitive block about the emotion to be expressed through facial expression—*(v,a)* values, the sentence to say, and the point to look at, are merged and continuously executed thanks to the actuation system, which translate them in movements performed by the motors that drive the face, the mouth and the neck of the ginoid^51^. Furthermore, the SEAI architecture is completely modular and portable, all the blocks composing the framework are stand-alone applications that process a limited set of information. These modules are distributed in a local net of computers that communicate by means of the YARP middleware (https://www.yarp.it/).This implies that each module can be activated or deactivated, and that the perception and cognitive systems can be used also without controlling the FACE Robot. As a result, we were able to use exactly the same rules engine in the computer box case, simply disabling the actuation part of the system that control the robot, and using instead the bluetooth speaker, presented as a smart computer box, actually running the same perception and actuation system of the robot. This led to a very close and controlled comparison.

### How the robot takes a decision, the rules engine

**Figure 5:**
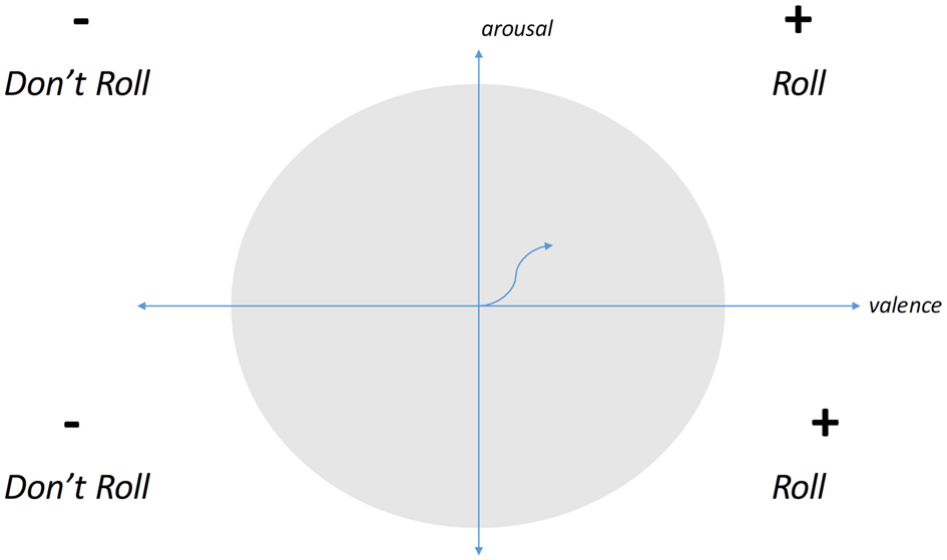
Decision Rule of the robot.

In this experiment, the robot (as well as the computer box) decides whether to *Roll* or *Don’t Roll* according to its emotional state and following its decision rules. In particular, a positive mood in SEAI (i.e., an emotional state with positive valence) will lead the robot to be collaborative with the human player and play *Roll*; while a negative mood in SEAI (i.e., an emotional state with negative valence) will lead the robot to play *Don’t Roll* (see Fig. 5). The decision is taken at the end of the interaction with Player-A, when the subject goes out of the room, and so out of the field of view of the robot.

If in the moment in which the robot has to take a decision, it is in a qualitatively neutral mood (v = 0, regardless the arousal), the decision will be taken randomly (50%). Participants’ behavior during all the time spent alone in the room with the robot, once observed by the Scene Analyzer and processed in SEAI, act as an input modifying the parameters of the robot which correspond to its ‘mood’, thus in turn affecting its course of action (i.e., its final decision). However, in this experiment, at each interaction with a new participant the robot always resetted its internal values at the <<neutral emotional state>> (which corresponds to *v* = 0, *a* = 0 in the graph). In conclusion, thanks to SEAI the robot was completely autonomous, by means of the rules everything was pre-programmed and automatized, starting from the rules that use perceived social cues to modulate the emotional state of the robot, to other rules determining which sentence it has to say, when to start and to end an interaction, and the storage of all the data acquired with timestamps in a structured dataset. The complete code of the rules engine is available upon request from the authors.

### Mean comparisons across groups

To compare the means (*μ*) of the distribution of a random variable for two independent groups (X, Y), we perform *t-Student* tests on the equality of means. Specifically, to test for *μ*_*x*_ = *μ*_*y*_ (when the variances *σ*_*x*_ and *σ*_*y*_ are unknown and replaced by *s*_*x*_ and *s*_*y*_) the test is $t=\frac{\bar{{x}}-{\bar{y}}}{(\frac{{s_{x}^{2}}}{n_{x}}+\frac{{s_{y}^{2}}}{n_{y}})^{1/2}}$ which is distributed as Student’s *t*. When the random variable is not continuous but a proportion, we use a normally distributed test statistic calculated as $z=\frac{{\hat{p_{x}}-\hat{{p_{y}}}}}{(\hat{p_{q}}(1-\hat{{p_{q}})(1/n_{1}+1/n_{2}}))^{1/2}}$ where $p_{x}=\frac{{x+y}}{n_{1}+n_{2}}$ where *x* and *y* are the number of successes in the two populations.

Both *t* and proportion tests rely on assumption about the distribution of the data. This is the reason why we also rely on permutation tests, which are nonparametric tests—i.e. do not rely on any assumption about the distribution of the data. Permutation tests work by resampling the observed data many times. The permutation test based on means implies: (1) to compute the sample means for each group $d_{observed}={\bar{x}}-{\bar{y}}$; (2) pool all the data together and randomly permute the pooled data; (3) then compute again the sample mean again for the two groups and note the difference *d*_1_; (4) repeat step 2 and 3 several times in order to obtain several mean differences, i.e. *d*_1_, *d*_2_, *d*_3…_. If the null hypothesis of no difference between the two groups is true, by changing the oder of the data we should not observe any difference in the means, otherwise it should look different from the real data. The ranking of the real test statistic, i.e. *d*_*observed*_, among the shuffled test statistics, *d*_1_, *d*_2_, *d*_3…_, gives a *p* value.

### A stylized behavioral model

We shall now modify our basic game to take into account that, prior to playing the trust game, Player B has sent a (cheap-talk) message containing either a promise to play *Roll* (‘promise’ conditions), or a generic message (‘empty’ conditions). It has been documented that humans are, to a lesser or greater extent, averse to lying (^37,38^). In our game, lying aversion can be represented by a cost of lying, *c*, that players incur in if their choice implies a lie.

**Figure 6:**
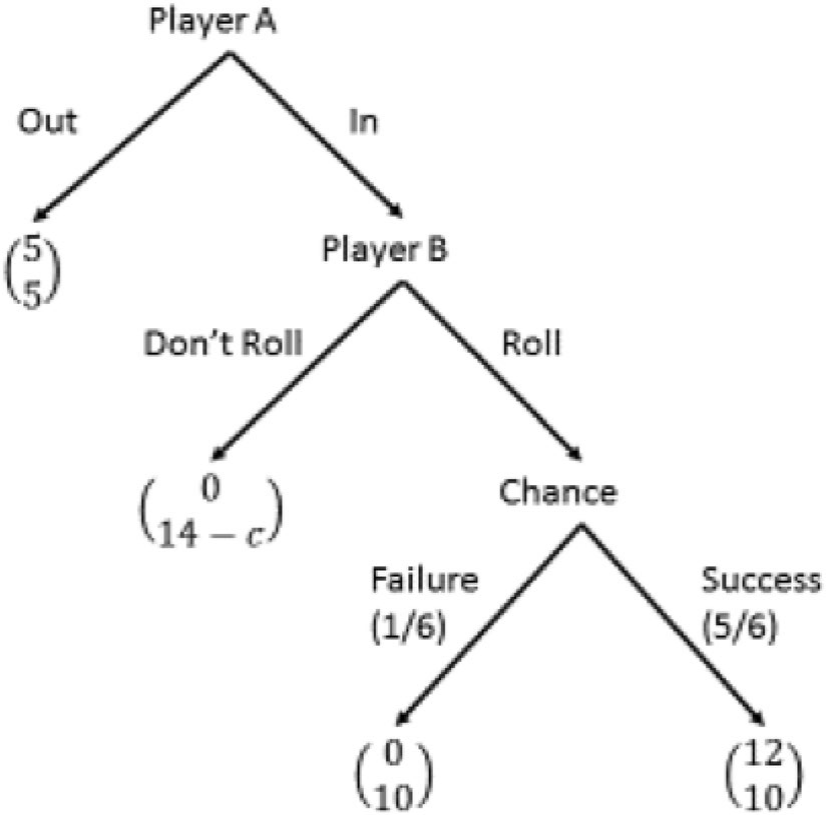
The trust game in the ‘promise’ conditions with lying aversion.

Note that in our experiment, the assumption of lying aversion would not have any effect in the ‘empty’ conditions, whereas it alters the payoff structure in the ‘promise’ conditions. In the ‘empty’ conditions the game form would still be represented by Fig. 2 and, in the unique (subgame perfect) equilibrium of the game, player-A chooses *Out* and player-B chooses *Don’t Roll*. However, in the ‘promise’ conditions, the payoff to Player-B from the choice *Don’t Roll* is reduced by the lying cost *c*. Thus, under the assumption of aversion to lying, in the ‘promise’ conditions the game is represented in Fig. 6.

If we now compute the (subgame perfect) equilibrium of the game in Fig. 6, we observe that the optimal choice of player-B depends on the value of *c*. If is low (*c* < 4), the optimal choice is as in the basic game (i.e., *Don’t Roll*—cf. Fig. 2) and, therefore, the promise has no effect. On the contrary, if the cost *c* is large enough (*c* > 4), the best choice for Player-B after sending a promise is to choose action *Roll* and, as a consequence, by anticipating this decision, Player-A optimally chooses action *In*.

Given that our experimental subjects played the role of Player-A, in order to rationalize their decisions in terms of this behavioral model, we need to conjecture on their beliefs on the value of *c*. Under the lens of this model, a subject may find it optimal to choose In if (s)he believes that Player-B has a lying cost that is high enough (or, more precisely, if (s)he attains a high probability to the fact that Player-B has enough aversion to lying). Since aversion to lying is eminently a human feature, a reasonable assumption is that the belief on cost depends on the degree of human-likeness of Player-B (as perceived by Player-A). In this respect, it can be the case that in the FACE condition, the higher the degree of human-likeness of FACE expressed by player-A, the higher the chance that a participant attains human characteristics (in our case, aversion to lying) to the humanoid, thereby reacting ‘as if’ interacting with a human. In other words, a higher human-likeness of Player-B could be associated to a higher expectation of Player-A on the cost *c*, and therefore, may rationalize the choice of *In*.

We note that, in our Human condition, the actress was instructed not to show any emotions when acting as Player-B, either facial or in the voice, in order to be more comparable to FACE. Thus, also in this case, the degree of human-likeness is a sensible measure. If we consider that the effect of human-likeness is likely to be relevant in the Human and FACE conditions (but not so much in Computer-box, in which participants just hear the message from a light-emitting audio-box), our simple behavioral model allows us to rationalize the fact that, both in the Human and FACE conditions with a ‘promise’, a higher human-likeness results in higher trust (i.e., choice of *In*).

### Description and analysis of physio data

Pulse rate variability (PRV) and electrodermal activity (EDA) signals are directly modulated by the autonomic nervous system (ANS) activity and, therefore, are considered ideal non-invasive physiological signals to investigate the ANS dynamics. Indeed, the ANS plays a crucial role in the processing of the emotional response, mental fatigue and workload^52–54^.

Particularly, the EDA signal measures the activity of eccrine sweat glands on the hand surface. Since sweat glands are directly innervated by the sympathetic branch of the ANS (and in particular the sudomotor nerve), the EDA analysis is considered one of the best ways to monitor the sympathetic activity^55^. As a preprocessing step, we applied the well-known cvxEDA model^56^ to remove the superimposed noise. From each free-to-noise EDA signal, we estimated the power spectrum within the frequency range of 0.045 and 0.25 Hz (EDAsymp), which has been demonstrated to be an effective estimator of the sympathetic nervous system activity^31^.

The PRV signal was computed interpolating the interbeat interval time series (IBI) extracted from the photoplethysmography signals acquired by the Empatica wearable acquisition system. To characterize the activity of the parasympathetic nervous system, which, as known, regulates the high frequency oscillations of the PRV signal, we estimated the Power Spectral Density (PSD) related to each PRV signal^32^. Two main spectral bands were considered: low frequency (LF) band (ranging between 0.04 and 0.15 Hz), and high frequency (HF) band (from 0.15 to 0.4 Hz). Then, the power spectrum in the HF band normalized to the sum of LF and HF power (HFnu) was computed to quantify the activity of the parasympathetic nervous system.

Note that all physiological indexes computed during the interaction with the agent were normalized for each participant by dividing them by the baseline value computed before the interaction phase

### New index from the sympathovagal assessment

Emotions regulation process modulates the sympathovagal balance^57,58^, which is considered a reliable marker of the human affective state. Previous studies have suggested that LF power spectrum can provide a quantitative marker of the sympathetic outflow and have used the LF/HF ratio as a correlate of the sympathovagal balance. However, the LF power is now regarded as a measure of both sympathetic and vagal tone, leading to ambiguities and possible inconsistent conclusions on the use of the LF/HF ratio as sympathovagal marker. In this study, we employed novel indexes of the sympathovagal dynamics based on the combination of the information extracted from the EDA and PRV signal^33^. Indeed, while EDAsymp reliably characterizes the sympathetic activity, HFnu is considered an effective cardiovascular-related features it that reliably quantify the parasympathetic outflow. Accordingly, we have estimated the sympathovagal balance using the ratio between EDAsymp and HFnu: EDAsymp/HFnu^33^.
